# A corpus-based analysis of the stylistic features of Chinese and American diplomatic discourse

**DOI:** 10.3389/fpsyg.2023.1122675

**Published:** 2023-02-14

**Authors:** Chenxia Zhang, Muhammad Afzaal, Abdulfattah Omar, Waheed M. A. Altohami

**Affiliations:** ^1^School of Languages, Shanghai University of International Business and Economics, Shanghai, China; ^2^Institute of Corpus Studies and Applications, Shanghai International Studies University, Shanghai, China; ^3^Department of English, College of Science & Humanities, Prince Sattam Bin Abdulaziz University, Al-Kharj, Saudi Arabia; ^4^Faculty of Arts, Port Said University, Port Said, Egypt; ^5^Department of Foreign Languages, Faculty of Education, Mansoura University, Mansoura, Egypt

**Keywords:** multi-dimensional analysis, diplomatic discourse, stylistic features, corpus studies, China and the United States

## Abstract

The study investigates the linguistic aspects of Chinese and American diplomatic discourse using Biber’s theoretical underpinnings of multi-dimensional (MD) analysis. The corpus of the study comprises texts taken from the official websites of the Chinese and US governments from 2011 to 2020. The study results show that China’s diplomatic discourse falls into the text type of *learned exposition* which includes informational expositions focused on conveying information. In contrast, the United States diplomatic discourse falls into the text type of “involved persuasion,” which is persuasive and argumentative. Furthermore, the two-way ANOVA test reveals few distinctions between spoken and written diplomatic discourse from the same country. Furthermore, T-tests demonstrate that the diplomatic discourse of the two countries differs significantly in three dimensions. In addition, the study highlights that China’s diplomatic discourse is informationally dense and context independent. In contrast, the United States diplomatic discourse is emotive and interactional, strongly dependent on context, and created within time restrictions. Finally, the study’s findings contribute to a systematic knowledge of the genre aspects of diplomatic discourse and are helpful for more effective diplomatic discourse system creation.

## Introduction

Diplomatic discourse, which belongs to institutional political discourse, refers to the discourse and discursive practices that “are used by sovereign states to communicate their international strategies and foreign policies in a certain historical period” (Jin, 2007, p. 21). It embodies “a country’s positions on cultures, ideologies, core interests, and strategic directions” (Du and Mei, 2019, p. 65). It mainly covers official diplomatic documents, national leaders’ speeches, interstate treaties, agreements, communique, declarations, statements, and press conferences (Afzaal et al., 2019, 2022). As the most official and authoritative way of international communication, diplomatic discourse reflects the government’s diplomatic efforts and policies, “showing the country’s attitudes and stances towards international communication” (Liu and Yang, 2019, p. 6; Niu and Relly, 2021). Diplomatic discourse’s essence is safeguarding and striving for national interests (Wang, 2008). Countries deal with international affairs with diplomatic discourse, through which international public opinion can be influenced, support from international communities can be gained, and discourse power in the international community can be improved.

Given the great significance of diplomatic discourse to not only diplomacy but also national development, studies of diplomatic discourse have become increasingly interdisciplinary and have drawn the attention of scholars from such various fields as linguistics, translation studies, communication, international studies, politics, and sociology (Hu and Tian, 2018; Liang, 2019; Tungkeunkunt and Phuphakdi, 2018; Yang and Zhou, 2020; Liu and Afzaal, 2021). However, studies of linguistic features of diplomatic discourse mainly focus on the relative distribution of linguistic features considered individually, despite the growing evidence that sets of co-occurring features can better reveal the underlying structure of textual variation (Biber, 1988, 2006). Therefore, a systemic analysis of the co-occurring linguistic features in the diplomatic discourse of China and the United States will offer valuable insights into how linguistic practices play a role in diplomacy.

Therefore, the study aims to compare the co-occurring linguistic features between China’s and the United States diplomatic discourse using the multi-dimensional (MD) analysis (Biber, 1988, 1995) approach. MD analysis is well suited for exploring differences in systemic textual variations between different groups of texts at the macro level. However, it has yet to be fully exploited in previous studies of diplomatic discourse.

The study of diplomacy has taken a linguistic turn since the 1990s, which has caught the interest of academics both domestically and internationally. As a result, the majority of research on diplomatic discourse focuses on its linguistic characteristics (Li and Hu, 2019; Liu et al., 2019), how the diplomatic discourse system is built (Sun, 2019; Fenton-Smith, 2020), how diplomatic discourse is translated (Fu, 2016; Yu and Wu, 2018), and how diplomatic discourse is disseminated (Tungkeunkunt and Phuphakdi, 2018; Yang and Zhou, 2020).

Previously, studies of linguistic features of diplomatic discourse are mainly carried out from the perspective of the relative distribution of linguistic features considered individually, such as nominalization, evaluation, negation, and some particular verbs (Li and Hu, 2013). di Carlo (2017) explores the role of the vagueness of the main modal verbs *shall*, *should*, and *may* in the institutional discourse of the UN and finds that these specific verbs in diplomatic discourse have double-faced strength in diplomacy. As a meaningful rhetorical device that carries speakers’ underlying thoughts, metaphors in diplomatic discourse have always been a priority in studies of linguistic features of diplomatic discourse (Wageche and Chi, 2017). Weng (2013) adopts corpus-based critical discourse analysis to compare metaphors used by the leaders from the UK, Canada, and China in their speeches at climate change talks and analyzes how these parties construct their identities through ideologies in metaphors. These studies signify the role that diplomatic discourse plays in the discursive construction of the ascribed identities of various countries (Liu and Wang, 2019) and the importance of the construction of diplomatic discourse to improve discursive power in the international community (Sun, 2019).

Considering the crucial role of translation and media in international communication, researchers in translation and communication contribute to the study of diplomatic discourse. Zanettin (2016) discusses the linguistic features of the source and translated diplomatic statements and argues that translation strategies of linguistic features of diplomatic discourse adopted by the media contribute to actively shaping international relations.

Many scholars examine the disseminative effects of diplomatic discourse to explore the relationship between diplomatic discourse and international relations (Tungkeunkunt and Phuphakdi, 2018). However, there are several noteworthy gaps in the existing literature. First, despite the growing popularity of corpus tools in linguistics, they are rarely used to analyze the linguistic features of diplomatic discourse. The corpus approach, which provides many authentic linguistic resources, is a valuable way to improve the representativeness and systematicness of linguistic features of diplomatic discourse studies. Second, studies have yet to compare linguistic variation in the diplomatic discourse of different countries. The way to gain more support in international affairs and earn a favorable reputation among international audiences can be further improved by comparing the linguistic features of the diplomatic discourse of different countries. Third, given the prominence of the discourse analysis perspective and the corpus-based approach in studies of the linguistic features of diplomatic discourse, MD analysis is well-suited for this type of research. Nevertheless, few existing studies have used this approach.

In addition, many studies have adopted different registers, such as academic discourse (Biber, 2003; Gray, 2015), legislative discourse (Cheng, 2012), and web discourse (Grieve et al., 2010). In addition to the synchronic perspective (Gray, 2015; Thompson et al., 2017), some studies take a diachronic perspective to investigate linguistic variation (Biber and Conrad, 2001; Westin and Geisler, 2002). Besides English registers, MD analysis has also been used to analyze non-English registers (Biber, 1995; Sardinha et al., 2014). The application of the MD approach in extensive registers and languages highlights the need for “further research in specialized registers and for greater attention to corpus design” (Pan, 2016). Meanwhile, these studies suggest that MD analysis can be applied to uncover the linguistic variation in the diplomatic discourse of China and the US and that findings on such variation will have practical implications for constructing a diplomatic discourse system. Through MD analysis, within the extracted dimensions, the meanings of groups of texts can be further interpreted to gain a more comprehensive understanding of the variation that is present in the corpus since it is necessary to go from text to context for a comprehensive and critical view of discursive practices (Bhatia, 2008).

The present study intends to apply this approach to uncover cross-cultural linguistic variations between China’s and the United States’ diplomatic and addresses the following three research questions:

1. Do countries and modes have similar effects on dimensions of linguistic variation in China’s and the United States’ diplomatic discourse?
2. How do China’s and the United States’ diplomatic discourse differ regarding linguistic features?
3. What are the similar dimensions of linguistic variation in China’s and the United States’ diplomatic discourse?

## Data and methods

### Multi-dimensional analysis

The study employs Biber’s theoretical underpinnings of multidimensional analysis as a theoretical framework. Based on the idea that “registers are best described concerning patterns of linguistic co-occurrence,” this method creatively uses factor analysis to reveal patterns of linguistic variation across registers (Biber et al., 2016, p. 646). This method has helped people gain “a sounder understanding of the most significant linguistic and nonlinguistic factors that influence register variation in English” (Nini, 2019). This analysis method is mainly applied to a corpus of texts representing various registers. First, the normalized frequency counts of individual features in the corpus that have the potential to distinguish between various registers are examined. Then, co-occurring features are sorted into “dimensions” using the statistical factor analysis method. The final qualitative interpretation of the resulting dimensions is “underlying functional associations” (Biber et al., 2016, p. 646), which states that linguistic features co-occur in the texts because they serve the same communicative purposes.

### Analysis procedure

The corpus is analyzed using Nini's (2015) Multidimensional Analysis Tagger (MAT), a computer program that analyses corpora or a single text using the multi-dimensional model put forth by Biber (1988). The accuracy of MAT to essentially replicate Biber’s findings has been demonstrated by research. The six dimensions Biber (1988) proposed as the foundation of MAT represent patterns of co-variation of 67 linguistic features and can explain linguistic variation in the most important English language registers (Nini, 2019). MAT uses Stanford Tagger first to annotate the linguistic features of the texts in the corpus, notably part of speech (POS; Toutanova et al., 2003). The dimension scores are then calculated using the normalized frequency counts of all linguistic features across 100 words.

This study compares Chinese and American diplomatic discourse’s linguistic features. The statistical analysis was conducted using MAT statistical data, and Rstudio (Version 4.0.5) was used. ANOVAs were used to compare written and spoken diplomatic discourse in the two sub-corpora to answer the first research question. For the second and third research questions, independent-sample t-tests were used to determine if and how diplomatic discourse in China and the US differ along the six functional dimensions. Then, AntConc compared linguistic feature frequencies. Finally, the functional significance of each dimension was determined by qualitatively comparing the two sub-corpora.

### Corpus of the study

A self-built corpus of Chinese and American diplomatic discourse was created to compare the linguistic characteristics of the two nations’ diplomatic discourses. The data were downloaded from the official websites of the Chinese and US governments, specifically the People’s Republic of China’s Ministry of Foreign Affairs and the United States Department of State from the period 2011 to 2020. The United States Department of State guides the United States on foreign policy matters and negotiates treaties and agreements with other countries. At the same time, the People’s Republic of China’s Ministry of Foreign Affairs manages foreign affairs and carries out China’s foreign policy. Therefore, the duties of the two departments and the information on their official websites are comparable, which ensures that the texts in this corpus can be compared. These texts, however, are used to exchange diplomatic messages and international strategies with other nations, which complies with the definition of diplomatic discourse provided at the beginning of the paper.

These texts were divided into written and spoken texts according to the classifications on the official websites. The written texts cover documents on important diplomatic issues, diplomatic activities, communique, agreements, foreign policies, and statements. In contrast, the spoken texts include the remarks and speeches of leaders of the countries and spokespersons of the Ministry of Foreign Affairs of the People’s Republic of China and the United States Department of State.

All texts were carefully processed after being downloaded from the websites, implying that garbled marks such as “&” and “^” were removed with the help of EditPad Pro. Finally, a corpus of 21,287,426 tokens, including 9,798 texts, was compiled (see Table 1). As for the sub-corpus of China’s diplomatic discourse (hereafter the China sub-corpus), there were 2,216,976 tokens and 2,082 texts in total, including 575,827 tokens in written texts and 1,641,149 tokens in spoken texts. About the sub-corpus of the United States diplomatic discourse (hereafter the US sub-corpus), there were 19,070,450 tokens in total, including 13,234,759 tokens in written texts and 5,835,691 tokens in spoken texts.

**Table 1:** Information on the corpus of China’s and the United States’ diplomatic discourse.

	China’s diplomatic discourse	United States’ diplomatic discourse	Total
Written	575,827 (909 texts)	13,234,759 (2,562 texts)	13,810,586 (3,471 texts)
Spoken	1,641,149 (1,173 texts)	5,835,691 (5,154 texts)	7,476,840 (6,327 texts)
Total	2,216,976 (2,082 texts)	19,070,450 (7,716 texts)	21,287,426 (9,798 texts)

## Results

The study compares the linguistic features of China’s and the United States’ diplomatic discourse according to Biber’s (1988) MD analysis. It finds that the differences of countries exert more influence on the six dimensions of linguistic features than that of modes of diplomatic discourse. With regards to countries, China’s and the United States’ diplomatic discourse differs significantly in terms of information density (D1), contextual dependence (D3), and the degree of elaboration of information generated under strict time constraints (D6). Additionally, the diplomatic discourse of the two countries shares some common features, such as non-narrative (low D2 scores), non-explicit in expressing the author’s point of view (intermediate D4 scores), and a mixture of the abstract and technical text type and the non-abstract text type (intermediate D5 scores). In general, China’s diplomatic discourse is closest to learned exposition in terms of its linguistic features, while the United States’ diplomatic discourse is similar to involved persuasion.

### Influences of countries and modes on dimensions of linguistic variation in China’s and the United States’ diplomatic discourse

Since there are two main factors in this study, namely, countries (China and the United States) and modes of diplomatic discourse (written and spoken), we conduct two-way ANOVA tests to determine if countries and modes of diplomatic discourse impact the six dimensions of linguistic features. The results of two-way ANOVA tests (Table 2) show that the factor of countries of diplomatic discourse exerts a more significant (*p* 0.05) impact on most dimensions (D1, D3, and D6), and the influences of both factors are significant on D2 and D5. Only on D4, the factor of modes of diplomatic discourse is more statistically significant than the factor of countries.

**Table 2:** Results of two-way ANOVA tests.

Dimension	Factor	*F*-value	*p*-value
Dimension 1 (D1) Involved vs. Informational Discourse	Country	400.889	<2e-16(***)
	Mode	3.237	0.0802
Dimension 2 (D2) Narrative vs. Non-Narrative Concerns	Country	5.549	0.0239(**)
	Mode	4.35	0.044(**)
Dimension 3 (D3) Context-Independent Discourse vs. Context-Dependent Discourse	Country	451.075	<2e-16(***)
	Mode	3.247	0.0797
Dimension 4 (D4) Overt Expression of Persuasion	Country	0.363	0.550264
	Mode	13.216	0.000839(***)
Dimension 5 (D5) Abstract vs. Non-Abstract Information	Country	4.288	0.04542(**)
	Mode	9.073	0.00466(***)
Dimension 6 (D6) On-Line Informational Elaboration	Country	204.74	<2e-16(***)
	Mode	0.02	0.889

**p < 0.005, ***p < 0.001.

Specifically, D4 deals with the degree of persuasion of the discourse, either “explicitly marking of the speaker’s persuasion (the speaker’s point of view) or argumentative discourse designed to persuade the addressee” (Biber, 1988). The significance of modes on D4 implies that the persuasion of diplomatic discourse is more about the differences between spoken and written discourse rather than the discourse of different countries. Apart from D4, the differences in linguistic features of diplomatic discourse of different countries are more significant than that of different modes. Generally speaking, there are few differences between spoken and written diplomatic discourse of the same country. Thus, in the following sections, the emphasis is put on exploring the coexistence of commonalities and differences in linguistic features of China’s and the United States diplomatic discourse, regardless of their modes.

### Differences in linguistic features between China’s and the United States’ diplomatic discourse

Table 3 summarizes the dimension scores of China and the US sub-corpora, alongside the results of the independent-sample t-tests for the dimension scores of the two sub-corpora. The diplomatic discourse in the two sub-corpora differs significantly in their scores on D1, D3, and D6 (*p* < 0.001) but not on D2, D4, and D5. As suggested by the results of the MD analysis using MAT, among eight text types for English found by Biber (1988), China’s diplomatic discourse is linguistically similar to learned exposition in terms of six dimensions, while the closest text type of the United States’ diplomatic discourse is involved persuasion. The former includes informational expositions focused on conveying information, with official documents, press reviews, and academic prose as typical registers. The latter includes persuasive and argumentative discourse represented by such registers as spontaneous speeches, professional letters, and interviews.

**Table 3:** Dimension scores and independent-sample *t*-tests results for the dimension scores of the two subcorpora.

Dimension	The China sub-corpus		The US sub-corpus		*t*-test	*p*-value
	Mean	SD	Mean	SD		
D1	−22.65	3.50	−0.46	3.71	−19.46	< 2.2e-16
D2	−3.32	0.65	−2.97	0.23	−2.26	0.03
D3	14.45	2.34	3.15	0.72	20.64	3.79e-16
D4	−0.22	1.73	−0.01	0.66	−0.53	0.60
D5	−0.73	1.04	−1.17	0.20	1.88	0.07
D6	−1.74	0.73	1.00	0.43	−14.50	2.50e-15

**Figure 1:**
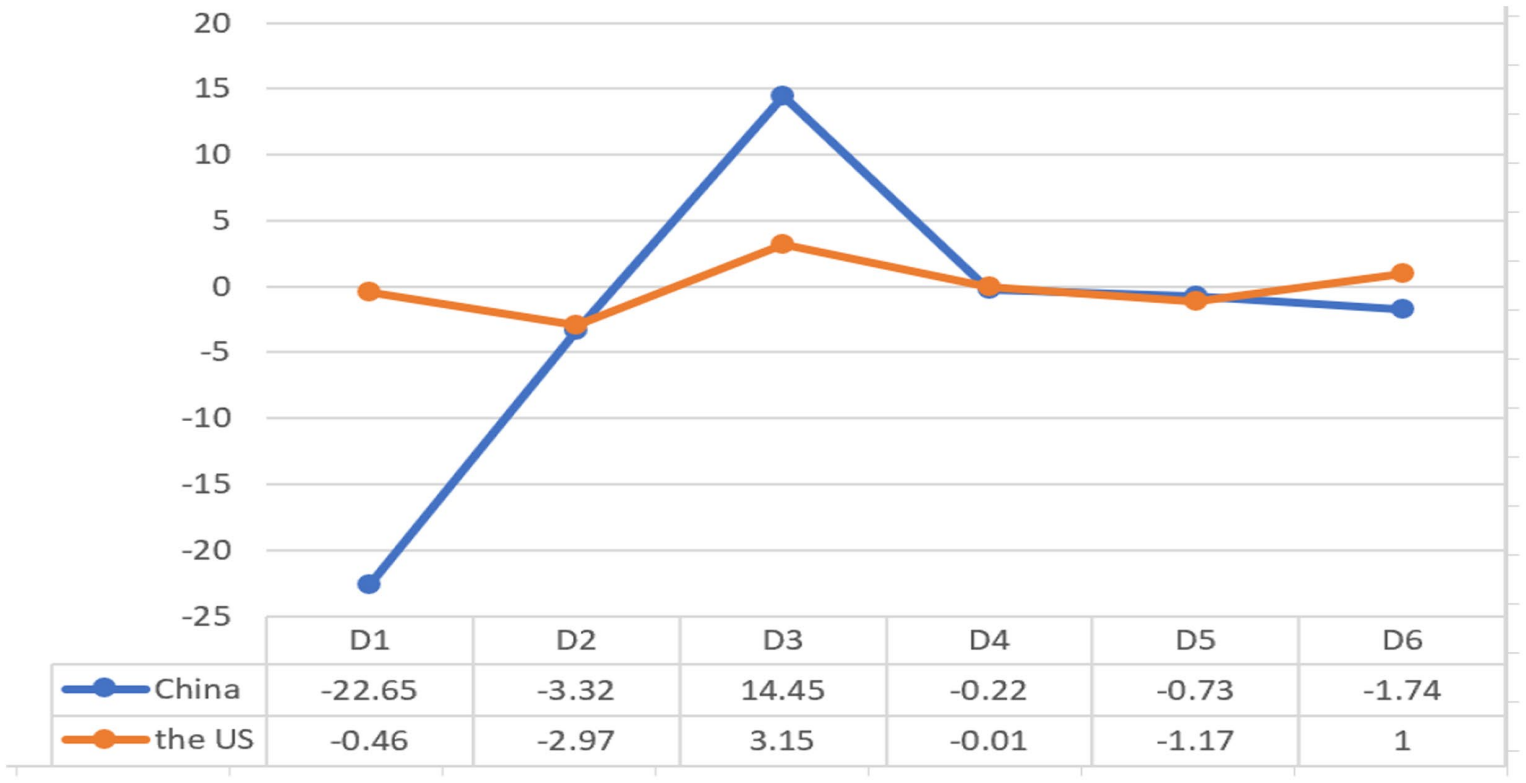
Differences in dimensions between the two sub-corpora.

Figure 1 shows that Dimension 1 marks high informational density and exact informational content versus affective, interactional, and generalized content (Biber, 1988), and presents linguistic features in interpersonal communication. The dominant features include many verb categories and complement clauses co-occurring with personal pronouns and past tense verbs on the positive side; while nouns, nominalizations, and adjectives appear on the negative side. There is a significant difference in D1 scores between China and the US sub-corpora (*t* = −19.46, *p* < 2.2e–16). The D1 score for the China sub-corpus (−22.65) is much lower than that for the US sub-corpus (−0.46). The lower the score on this Dimension, the denser the information in discourse. T-tests for the z-scores of these dominant features indicate that the two sub-corpora are different in 28 of the 34 features in Dimension 1. Due to limited space, Table 4 shows features that are significantly different (*p <* 0.05) between the two sub-corpora and have a weight larger than 0.80 in Dimension 1.

**Table 4:** Linguistic features that are significantly different between the two sub-corpora in Dimension 1.

Linguistic features	Mean		*t*-test	*p*-value
	China	US		
Private verbs	−0.9585	−0.351	−10.87	3.74e-13
*That* deletion	−0.6045	−0.0025	−18.833	< 2.2e-16
Present tense	−1.311	−0.19	−12.77	1.80e-12
Second person pronouns	−0.6425	0.1775	−33.133	< 2.2e-16
Pro-verb *do*	−0.792	−0.186	−28.567	< 2.2e-16
Total other nouns	3.38	2.091	2.5131	0.01921

Table 4 shows that the China sub-corpus has more nouns than the US corpus. Biber argues that since nouns are the main carriers of the referential meaning in the text, the high frequency of nouns is associated with “a high informational focus and a careful integration of information in a text” (Biber, 1988, p. 104). Accordingly, China’s diplomatic discourse has a greater density of information. Private verbs and present tense indicate a verbal style. Present tense refers to actions occurring in the immediate context of interaction, and private verbs (e.g., *think*, *feel*) are used for the overt expression of private attitudes, thoughts, and emotions (Biber, 1988). Meanwhile, second-person pronouns are frequently used in highly interactive discourse. In addition, *that*-deletion and pro-verb *do*, which represent a reduction in surface form, result in a more generalized, uncertain content. These linguistic features appear more frequently in the United States’ diplomatic discourse than in China’s diplomatic discourse, implying that the former has the feature of higher interpersonal interaction and higher expression of personal feelings compared to the latter. The dense information of China’s diplomatic discourse is partly reflected in its high frequency of nouns, which is partially related to a lower frequency of first-person and second-person pronouns. Specifically, instead of using *we* for self-reference, China frequently uses *China* in its diplomatic discourse, even in spoken texts (Example 1). The United States uses more private verbs that express overt expression of attitudes and thoughts (Example 2), which makes the information in the US sub-corpus more affective, interactional, and generalized.

#### Example 1

At the international area, China is not known for bullying the weak. China has only been commended for speaking out for developing countries in defiance of the bullying by strong powers. That is why China has been winning friends all over the world.

#### Example 2

I think there are a number of areas of cooperation in terms of information sharing so we can track individuals, border security so that hopefully we can keep these individuals from returning, cooperation around extremists and terror financing networks and creating safe havens for terrorists. So I think, again, a significant amount of work to do within the East Asian Ministers discussions as well.

Dimension 3 distinguishes between highly explicit, context-independent discourse and nonspecific, situation-dependent discourse, covering five linguistic features with large positive weights and three with large negative weights. High scores on this dimension suggest that the discourse does not depend on context for interpretation (Biber, 1988). Both sub-corpora have positive scores on this dimension, which are also the highest among the six dimension scores, showing a strong feature of independence from context. Nonetheless, the dimension score of China sub-corpus is significantly higher than that of the US sub-corpus, and the two sub-corpora differ in all the linguistic features in this dimension (Table 5).

**Table 5:** Linguistic features that are significantly different between the two subcorpora in Dimension 2.

Linguistic features	Mean		*t*-test	*p*-value
	China	US		
WH relative clauses on object position	−0.802	−0.7275	−8.9221	8.78e-11
Pied-piping relative clauses	−0.439	−0.2475	−5.9826	7.67e-07
WH relative clauses on subject position	−0.7775	−0.355	−8.7456	3.96e-10
Phrasal coordination	8.6795	1.344	22.026	< 2.2e-16
Nominalizations	3.0785	0.938	9.047	1.21e-08
Time adverbials	−0.9925	−0.021	−14.99	5.85e-14
Place adverbials	−0.48	−0.0895	−6.5562	7.62e-07
Total adverbs	−2.794	−1.8385	−10.782	1.43e-10

Phrasal coordination and nominalizations occur much more frequently in China’s diplomatic discourse, while WH relative clauses on object positions, WH relative clauses on subject positions, pied-piping constructions, time adverbials, place adverbials, and adverbs, are used slightly more frequently in the United States’ diplomatic discourse. Accordingly, China’s diplomatic discourse is more context-independent than the United States’ diplomatic discourse by using phrasal coordination and nominalizations (Example 3), which are devices for idea unit expansion and informational integration (Chafe, 1982; Chafe and Danielewicz, 1986, to pack information into a text in a less context-dependent way by omitting context-dependent information such as the agent, time, and location. That the United States’ diplomatic discourse is highly explicit, elaborated, and endophoric is embodied in its use of three different forms of relative clauses (Example 4), which pack information into noun phrases instead of expressing the information as separate and independent clauses. Though the two sub-corpora have different linguistic features, their marking of referents in an elaborated and explicit manner echoes the genre of diplomatic discourse.

#### Example 3

China is promoting industrial upgrading through scientific and technological innovation, advanced industrialization, IT application, urbanization and agricultural modernization all at the same time, pursuing balanced and mutually reinforcing development between regions, and pushing for integrated urban–rural development.

#### Example 4

One of the biggest is the Foundation for the Future, which is based in Jordan and which is an independent NGO that supports civil society development throughout the BMENA region.

Dimension 6 deals with the elaboration of information generated under strict time constraints, leading to fragmentation of the presentation of information accomplished by tacking on additional dependent clauses, or an integrated presentation that packs information into fewer constructions containing more informative words and phrases (Biber, 1988). In general, the dimension score of the US sub-corpus is significantly higher than that of the China sub-corpus, and they have different linguistic features in this dimension (Table 6), indicating that the China sub-corpus contains significantly fewer features of texts produced online. The lower dimension score of China sub-corpus is characterized by limited use of *that*-clauses (including *that* complements to verbs or adjectives and *that* relative clauses on object positions), demonstratives, and existential *there*. The significant differences between these linguistic features indicate that though the diplomatic discourse of both countries represents a formal, planned type of discourse, the United States’ diplomatic discourse is more likely to use *that*-clauses and demonstratives than China’s diplomatic discourse, to expound on international affairs that could otherwise be described with denser information, as evidenced by Example 5. Both countries’ diplomatic discourse has an informational focus, but the United States’ diplomatic discourse shows the feature of being produced under real-time conditions compared to China’s diplomatic discourse. The latter is associated with more formal and planned discourse.

**Table 6:** Linguistic features that are significantly different between the two sub-corpora in Dimension 6.

Linguistic features	Mean		*t*-test	*p*-value
	China	US		
*That* verb complements	−0.4535	0.092	−6.5749	1.60e-07
Demonstratives	−1.287	0.9065	−17.965	< 2.2e-16
*That* relative clauses on subject position	0.519	3.232	−21.758	< 2.2e-16
*That* adjective complements	−0.089	0.95	−9.8172	1.27e-11
Total prepositional phrases	0.3895	−0.846	10.014	2.35e-10
Existential *there*	−0.909	0.383	−10.161	1.98e-10
Demonstrative pronouns	−0.6215	0.676	−17.938	< 2.2e-16
WH relative clauses on subject position	−0.7775	−0.355	−8.7456	3.96e-10
Phrasal coordination	8.6795	1.344	22.026	< 2.2e-16

#### Example 5

But we have a lot more going on across the region, and this trip is really an opportunity to showcase that these other dimensions of U.S. engagement in the Middle East and in the Gulf, particularly the emphasis that we have placed on building partnerships beyond the government-to-government level.

### Similar dimensions of linguistic variation in China’s and the United States’ diplomatic discourse

The results of independent-sample t-tests show no statistically significant differences in the dimension scores of China’s and the United States’ diplomatic discourse on Dimensions 2, 4, and 5.

Low D2 scores indicate that the texts in the corpus are non-narrative, belonging to static, descriptive or expository types of discourse. The D2 scores for the China and the US sub-corpora are both low, demonstrating the non-narrative feature of diplomatic discourse. Table 7 shows that the two subcorpora have limited use of perfect aspect, public verbs, and synthetic negation. All these linguistic features represent narrative action in texts. Specifically, perfect aspect is used to describe past events; public verbs are often markers of indirect and reported speech commonly used to introduce indirect statements; synthetic negation is one of the depictive details. The limited use of these features of narrative discourse indicates that both countries place more emphasis on discussing current efforts and future development rather than past achievements (Example 6). They do not tend to “express intellectual states (e.g., believe) or nonobservable intellectual acts (e.g., discover)” (Biber, 1988, p. 96). Meanwhile, it also implies that diplomatic discourse is often marked by non-narrative concerns, whether expository or descriptive.

**Table 7:** Linguistic features of the two sub-corpora in Dimension 2.

Linguistic features	Mean		*t*-test	*p*-value
	China	US		
Past tense	−0.9355	−0.517	−9.662	3.74e-11
Third person pronouns	−1.11	−0.7695	−12.427	7.17e-15
Perfect aspect	−0.522	−0.559	0.23571	0.816
Public verbs	−0.425	−0.4695	0.30033	0.7669
Synthetic negation	−0.648	−0.48	−2.4881	0.01956
Present participial clauses	0.32	−0.1765	4.0255	0.0004244
Present tense	−1.311	−0.19	−12.77	1.80e-12
Attributive adj.	1.786	−0.329	7.916	1.43e-07
Past participial WHIZ deletion relatives	−0.229	−0.583	6.1712	4.85e-06
Word length	2.2605	0.0505	17.484	4.46e-15

#### Example 6

The United States will continue to provide lifesaving assistance to the millions of Darfuris who are affected by this conflict until the humanitarian situation improves.

Table 7 shows that Dimension 4 concerns the degree to which persuasion is marked overtly, involving the overt marking of authors’ points of view and their assessment of the advisability or likelihood of an event presented to persuade the audience (Biber, 1988). The D4 scores of both sub-corpora are not high, implying that the diplomatic discourse in the present study does not show explicit marking of persuasion to its audience. Independent-sample t-tests reveal that there are no significant differences in the use of predication models (e.g., will/would/shall) and suasive verbs (e.g., command, stipulate) between the two sub-corpora (Table 8). The former presents direct statements that some events will occur, while the latter indicates the intention of something to happen in the future. Though the use of these linguistic patterns that signify overt markers of persuasion is limited in both countries’ diplomatic discourse, the two sub-corpora have intermediate scores on this dimension, indicating that the genres of the two sub-corpora are relatively undistinguished on this dimension.

**Table 8:** Linguistic features of the two sub-corpora in Dimension 4.

Linguistic features	Mean		*t*-test	*p*-value
	China	US		
Infinitives	0.569	1.338	−3.6596	9.84e-04
prediction modals	0.6675	0.0945	3.1416	0.005014
Suasive verbs	0.2575	0.1675	0.54565	0.5915
Conditional adverbial subordinators	−1.026	−0.0235	−17.05	1.44e-15
Necessity modals	0.346	−0.551	4.9821	7.02e-05
Split auxiliaries	−1.034	−1.028	−0.048144	9.62e-01
Possibility modals	−1.098	−0.297	−13.142	1.04e-15

Dimension 5 distinguishes between highly abstract, technical discourse, and non-abstract types of discourse. Both sub-corpora have low values on this dimension, implying that the texts in the corpus convey information in a non-abstract way. It can be found from Table 9 that there are no significant differences in the use of adverbial subordinators and passive clauses, including agentless passives and by-passives, between China’s and the United States’ diplomatic discourse, suggesting that both sub-corpora do not make frequent use of these linguistic features that mark informational discourse that is abstract. Passive constructions are frequently used to highlight the patient of the verb, the entity acted upon, which is usually an inanimate referent and is often an abstract concept rather than a concrete referent (Biber, 1988). Adverbial subordinators co-occur with passive forms to mark the complex logical relations among clauses. Since passive forms frequently appear in abstract and technical texts with formal style, the findings show that these two sub-corpora do not have the feature of the frequent use of these co-occurring features. Likewise, the mean of other linguistic features in this dimension is close to 0, indicating a mixture of two content types, namely, the abstract and technical one and the non-abstract one.

**Table 9:** Linguistic features of the two sub-corpora in Dimension 5.

Linguistic features	Mean		*t*-test	*p*-value
	China	US		
Conjuncts	0.01	−0.061	0.70985	4.83e-01
Agentless passives	−0.7315	−0.5965	−2.69	0.01353
Past participial clauses	0.7625	0.05	4.504	0.0001667
By-passives	−0.1425	−0.2035	1.3103	0.2025
Past participial WHIZ deletion relatives	−0.229	−0.583	6.1712	4.85e-06
Other adverbial subordinators	0.224	0.0675	1.3176	0.2026
Predicative adjectives	−0.6985	0.7945	−11.054	3.78e-11

## Discussion

The results show that the impact of different countries on dimensions of linguistic variation in diplomatic discourse is more significant than that of modes of discourse. As mentioned earlier, diplomatic discourse is a typical institutional political discourse produced under the government’s strict supervision. Accordingly, the consistency of linguistic features of the diplomatic discourse of a country is closely related to maintaining its credibility and authority in the international community, promoting the dissemination of its diplomatic philosophies, and gaining support in international affairs. Though general spoken and written discourse often demonstrates different linguistic features, the present study’s findings indicate that to highlight the solemn and formal characteristics of the diplomatic settings, spoken diplomatic discourse presents a large number of linguistic features of written diplomatic discourse. For instance, the spoken diplomatic discourse has law values on the linguistic features that best reflect colloquialism and interactivity, such as private verbs, second-person pronouns, contraction, logical negation, demonstratives, etc. In particular, the only dimension on which spoken and written diplomatic discourse differs significantly is D4, indicating that the overt expression of persuasion in spoken diplomatic discourse is more evident than that in written diplomatic discourse. Furthermore, since the spoken texts in the corpus are the remarks and speeches of leaders of the countries and spokespersons of the Ministry of Foreign Affairs of the People’s Republic of China and the United States Department of State, they tend to provide not only detailed information to the audience to fully express their views, but also use linguistic devices such as prediction models, suasive verbs, and necessity modals, to justify their arguments so that the audience can accept their points of views (Chong and Druckman, 2007; Li, 2014)). Accordingly, in diplomatic discourse, spoken discourse demonstrates many linguistic features typically and exclusively belonging to written discourse.

Diplomatic discourse of different countries often presents different linguistic features on account of their different national positions and national interests in international affairs. For instance, a developed country may hope to maintain its monopoly in certain fields through diplomacy, while developing countries may need international cooperation and support from other countries to strengthen their own development. Thus, while diplomatic discourse has some commonalities as a whole, there are differences in the diplomatic discourse of different countries. This feature is vividly presented in the present study. Namely, China and the United States have different ideologies and cultures, which party contribute to the differences in linguistic features of the diplomatic discourse of the two countries.

Regarding the second research question, the study shows that, regarding linguistic features, China’s diplomatic discourse is most similar to learned exposition. In contrast, the United States diplomatic discourse is closest to involved persuasion. They present significant differences in information density (D1), contextual dependence (D3), and the degree of elaboration of information generated under strict time constraints (D6). First, the information density of the China subcorpus is significantly higher than that of the US subcorpus. The difference in D1 scores is due to the greater emphasis of China’s diplomacy on providing more information in the conduct of international affairs by using more content words (mainly including nouns, verbs, adjectives, and adverbs) in diplomatic discourse. This is also evidenced by the findings of Hu and Tian’s (2018) research on the English translations of China’s diplomatic discourse, in which the authors point out that China’s diplomatic discourse uses content words frequently to project its image of being down-to-earth in diplomacy.

Second, the context dependency of the China subcorpus is significantly low, as indicated by its higher D3 score. Though both countries’ diplomatic discourse does not depend on context for interpretation, this feature is more pronounced in China’s diplomatic discourse, as evidenced by its significantly higher score on this dimension. To be specific, the key linguistic feature characterizing the high D3 score of China’s subcorpus is nominalization, which “allows the dense packing of complex ideas into elements of clause structure, the addition of modifiers and qualifiers, and the backgrounding and foregrounding of information in the discourse” (Jucker et al., 2015). In addition to the influence of the Chinese source language, the frequent use of nominalization largely depends on the diplomatic genre. In the case of Chinese, the structure in which the subject is omitted may trigger the use of nominalized structures in China’s diplomatic discourse (Hou, 2013). Since “the frequencies of English nominalization are directly related to the formalness of the text type in which it appears” (Wang, 2003, p. 74), the frequent use of normalization in the China subcorpus indicates that the text type of diplomatic discourse in China is severe and formal.

Third, the China subcorpus has significantly lower D6 scores, marking its independence from on-line elaboration strategies for the production of informational discourse (Biber, 1988). The less on-line information elaboration is always closely related to more shared background knowledge (Biber, 1988). In this case, the diplomatic discourse on the official websites of both countries is intended for professional and semi-professional readers who are expected to be interested in and have basic background knowledge of diplomacy. The lower D6 score of the China subcorpus suggests that China, represented by the Ministry of Foreign Affairs of the People’s Republic of China in handling international affairs, may have assumed more background knowledge than the United States, represented by the US Department of States. The typical linguistic features, including *that* verb complements and demonstratives, are useful tools for “the expression of opinions, attitudes, or personal statements of individuals” (Biber, 1988, p. 160). Accordingly, the high score of the US subcorpus may also echo its low D4 score, which suggests a low degree of explicitness in expressing the producers’ views.

In terms of the third question, though some differences in linguistic features of China’s and the United States’ diplomatic discourse are identified, there are also some features that do not differ significantly between the two sub-corpora, which may indicate the commonalities of diplomatic discourse and the consistent genre features. Both China’s and the United States’ sub-corpora have low D2 scores, signifying their non-narrative feature. Their low D2 scores mainly result from the strong tendency of countries to place greater emphasis on current international affairs and future developments rather than past achievements. In this way, both China and the United States, as two major countries, hope to strengthen their images as responsible countries in proactively handling international affairs and show their abilities to promote global development in the future to gain more support. Meanwhile, the limited use of public verbs, referring to markers of indirect and reported speech commonly used to introduce indirect statements, in diplomatic discourse reinforces the authority of diplomatic discourse.

Besides, the degree of persuasion is not high in both countries’ diplomatic discourse, as evidenced by their intermediate D4 scores, implying that diplomatic discourse is inexplicit in expressing the producers’ points of view. Vagueness is a typical feature of diplomatic discourse, which is often prominent in various diplomatic settings, especially the press conference of the Ministry of Foreign Affairs. International situations are complex and changeable, and uncertainty is the norm. The feature of vagueness of diplomatic discourse makes it possible for countries to implicitly express their national positions in handling international affairs (Nicholson, 1963). In this way, vague expressions in diplomacy can avoid the adverse effects of misjudgments, facilitate strategic adjustment after the situation changes, avoid direct conflicts, and maintain and improve international relations. The results of the present study confirm previous findings that national positions and diplomatic stances are often implicitly expressed by the producers of diplomatic discourse.

Finally, there is no significant difference in the degree of abstractness of diplomatic discourse between the two countries. However, the D5 score of the China sub-corpus is below zero, and that of the US sub-corpus is above zero. This suggests that diplomatic discourse needs to show a clear tendency to express viewpoints and positions in a technical, abstract, and formal way. Nevertheless, the two subcorpora are similar in this dimension in terms of content and style. The fundamental goal of diplomatic discourse is to make its audience understand the connotations of diplomatic discourse in handling international affairs. However, diplomatic discourse must be concrete. Otherwise, it will lose its authority. Accordingly, diplomatic discourse strikes a balance between being understandable to the audience and maintaining authority, resulting in a mix of abstract and non-abstract content types.

## Conclusion

The study explored Chinese and American diplomatic discourses using the multi-dimensional (MD) analysis proposed by Biber (1988). The study investigated the influences of countries and modes on linguistic variation in China’s and the United States’ diplomatic discourse. The study also presented the differences in linguistic features between the two countries’ diplomatic discourse. The results showed that China’s diplomatic discourse falls into the text type of “learned exposition,” emphasizing conveying information. In contrast, the United States diplomatic discourse belongs to the text type of “involved persuasion,” which is persuasive and argumentative.

The study identifies that the impact of different countries on dimensions of linguistic variation in diplomatic discourse is more significant than that of modes of discourse. Spoken diplomatic discourse exhibits many linguistic features usually associated with written discourse. By contrast, the diplomatic discourse of different countries often presents different linguistic features on account of their different national positions and national interests in international affairs.

The present study’s findings have functional, practical implications in dealing with international affairs. As the authoritative and official channel for countries to express their national positions on various foreign affairs, diplomatic discourse plays a pivotal role in constructing the diplomatic discourse system, the discursive construction of the national image, and enhancing discourse power in the international community. The differences in stylistic features between China’s and the United States’ diplomatic discourse revealed by the present study make it possible to understand the differences between the two countries regarding ideologies and cultures, diplomatic philosophies, and national conditions. Meanwhile, a comprehensive understanding of the typical linguistic features of diplomatic discourse contributes to a further understanding of the nature of diplomacy and the critical role of discourse in the construction of international order and handling of international affairs.

## Data availability statement

The raw data supporting the conclusions of this article will be made available by the authors, without undue reservation.

## Author contributions

All authors listed have made a substantial, direct, and intellectual contribution to the work and approved it for publication.

## Funding

This study is supported via funding from Prince Sattam Bin Abdulaziz University project number PSAU/2023/R/1444.

## Conflict of interest

The authors declare that the research was conducted in the absence of any commercial or financial relationships that could be construed as a potential conflict of interest.

## Publisher’s note

All claims expressed in this article are solely those of the authors and do not necessarily represent those of their affiliated organizations, or those of the publisher, the editors and the reviewers. Any product that may be evaluated in this article, or claim that may be made by its manufacturer, is not guaranteed or endorsed by the publisher.
